# STAIR-DETR: A Synergistic Transformer Integrating Statistical Attention and Multi-Scale Dynamics for UAV Small Object Detection

**DOI:** 10.3390/s25247681

**Published:** 2025-12-18

**Authors:** Linna Hu, Penghao Xue, Bin Guo, Yiwen Chen, Weixian Zha, Jiya Tian

**Affiliations:** 1 School of Network and Communication Engineering, Jinling Institute of Technology, Nanjing 211169, China; 2416901107@stu.jit.edu.cn (Y.C.); 2507010001@stu.jit.edu.cn (W.Z.); 2 School of Software Engineering, Jinling Institute of Technology, Nanjing 211169, China; 2407010018@stu.jit.edu.cn; 3 College of Computer and Information Engineering, Xinjiang Agricultural University, Urumqi 830052, China; gb@xjau.edu.cn; 4 School of Information Engineering, Xinjiang Institute of Technology, Aksu 843100, China; 2020032@xjit.edu.cn

**Keywords:** multi-scale semantic integration, real-time object recognition, small object detection, UAV imagery

## Abstract

Detecting small objects in unmanned aerial vehicle (UAV) imagery remains a challenging task due to the limited target scale, cluttered backgrounds, severe occlusion, and motion blur commonly observed in dynamic aerial environments. This study presents STAIR-DETR, a real-time synergistic detection framework derived from RT-DETR, featuring comprehensive enhancements in feature extraction, resolution transformation, and detection head design. A Statistical Feature Attention (SFA) module is incorporated into the neck to replace the original AIFI, enabling token-level statistical modeling that strengthens fine-grained feature representation while effectively suppressing background interference. The backbone is reinforced with a Diverse Semantic Enhancement Block (DSEB), which employs multi-branch pathways and dynamic convolution to enrich semantic expressiveness without sacrificing spatial precision. To mitigate information loss during scale transformation, an Adaptive Scale Transformation Operator (ASTO) is proposed by integrating Context-Guided Downsampling (CGD) and Dynamic Sampling (DySample), achieving context-aware compression and content-adaptive reconstruction across resolutions. In addition, a high-resolution P2 detection head is introduced to leverage shallow-layer features for accurate classification and localization of extremely small targets. Extensive experiments conducted on the VisDrone2019 dataset demonstrate that STAIR-DETR attains 41.7% mAP@50 and 23.4% mAP@50:95, outperforming contemporary state-of-the-art (SOTA) detectors while maintaining real-time inference efficiency. These results confirm the effectiveness and robustness of STAIR-DETR for precise small object detection in complex UAV-based imaging scenarios.

## 1. Introduction

Due to their maneuverability, low operational cost, and deployment efficiency, UAVs have been widely adopted in domains like traffic monitoring, emergency response, precision agriculture, forest inspection, urban management, and military surveillance [1,2]. As the low-altitude domain experiences expansion, UAVs are assuming increasingly critical roles in national infrastructure and urban management, thereby raising new demands for real-time visual perception in complex environments [3]. Nevertheless, the growing demand for real-time detection of small and dynamic targets introduces new challenges in processing aerial imagery captured during rapid low-altitude flights, in which extremely small targets (e.g., pedestrians, bicycles, early crop disease spots, or intruders in forests) must be detected with high accuracy and efficiency [4].

In standard UAV aerial settings, flight altitudes spanning from several dozen to hundreds of meters, combined with restricted sensor resolution, mean that objects in captured images usually occupy only a few pixels (often < 32 × 32), making them blurry, sparse, and highly susceptible to background interference. Furthermore, challenges such as motion blur, perspective distortion from camera pose variations, and lighting or atmospheric noise further complicate detection. UAVs also operate in complex backgrounds (e.g., urban textures, forest canopies, building shadows, water reflections), resulting in low contrast and increased false detections [5]. Therefore, it becomes essential to design detection algorithms that effectively preserve fine-grained object details while capturing global context, striking a compromise between performance in real time and accuracy.

Various approaches have been proposed to settle these issues. Early methods based on handcrafted features (e.g., Viola–Jones, HOG+SVM) offered real-time capability in constrained scenarios but lacked robustness to scale variation and background complexity [6,7]. Two-stage detection frameworks rooted in deep learning—like R-CNN [8] and its Faster R-CNN [9]—which enhance detection accuracy by integrating feature learning with region proposal mechanisms; however, their multi-step inference pipeline led to substantial computational overhead, restricting their applicability in real-time UAV tasks. One-stage detectors accelerated inference by reformulating detection as regression; however, their reliance on fixed anchors and grid structures often results in poor performance on small and sparse targets. Multi-scale feature aggregation techniques, such as FPN [10], PANet [11], and BiFPN [12], have alleviated issues of scale inconsistency by integrating hierarchical features, yet their fusion strategies remain hand-crafted and lack dynamic adaptability in complex scenes, particularly for small target detection in UAV imagery.

More recently, Transformer-based architectures have shown promise in visual detection. DETR [13] introduced global self-attention to eliminate the need for anchor boxes and NMS [14], enabling an end-to-end detection paradigm. Despite its theoretical elegance, DETR exhibits limitations including high computational cost, slow convergence, and inadequate modeling of fine-grained features. Follow-up models such as Deformable DETR [15] adopted sparse attention to alleviate these issues but still struggled with extremely small object detection. RT-DETR [16] incorporates decoupled intra- and cross-scale interactions and IoU-guided query selection, thereby achieving real-time performance with improved accuracy. However, it still exhibits critical limitations: the shallow AIFI module demonstrates limited responsiveness to small object features; fixed upsampling and downsampling operations lead to loss of spatial details; and the decoder exclusively leverages P3–P5 features, rendering them inadequate for accurate regression of objects smaller than 16 × 16 pixels.

In order to get beyond the constraints indicated above, this work presents STAIR-DETR, a multi-scale optimized variant of RT-DETR, offering the following principal innovations:

(1) To replace the less effective AIFI module in the neck, we introduce the Statistical Feature Attention (SFA) mechanism, a token-level statistical self-attention module. By exploiting feature statistics such as mean and variance, SFA emphasizes salient local regions, suppresses background interference, and improves the model’s sensitivity to small objects with negligible computational cost.
(2) Within the backbone, the conventional Basic Block is re-designed into Diverse Semantic Enhancement Block (DSEB). This module incorporates multi-branch pathways and dynamic convolutions to extract features under varied receptive fields, thereby enriching semantic representation while retaining spatial precision, which is critical for complex UAV imagery.
(3) A new resolution conversion strategy is devised by unifying Context-Guided Down sampling (CGD) and Dy Sample. Unlike conventional operators that treat down- and up-sampling separately, the Adaptive Scale Transformation Operator (ASTO) performs context-aware compression and content-adaptive reconstruction in a joint framework. This design ensures high-fidelity feature transmission across scales and mitigates detail loss, particularly for fine-grained small object cues.
(4) Aiming to improve the representation of extremely small targets, an extra P2 detection head is introduced in the Transformer decoder, leveraging high-resolution shallow features for accurate classification and regression, thereby enhancing detection performance for tiny targets.

In summary, the proposed improvement measures have been integrated into STAIR-DETR, which is a unified framework specifically designed for the detection of small objects in drone images. Specifically, the goal of STAIR-DETR is to address several key issues observed in actual drone images: (i) The targets are usually very small and densely distributed, resulting in the loss of shallow details after early downsampling; (ii) Objects have significant scale variations and frequent occlusions, so traditional feature pyramids often lose consistency across different scales, thereby causing detection omissions; (iii) The complex aerial background brings strong visual interference, requiring a more discriminative representation for small targets. Evaluations on the VisDrone2019 [17] dataset confirm its superior accuracy and robustness over existing approaches, demonstrating both effectiveness and practical potential. The remainder of this paper is organized as follows. Section 2 reviews related work on object detection and UAV-based applications. Section 3 describes the STAIR-DETR architecture and its core modules. Section 4 shows the experimental setup, ablation studies, and performance comparisons. Section 5 discusses the findings, and Section 6 concludes the paper with future directions.

## 2. Related Work

This section is divided into three subsections that review the current related work in object detection. Section 2.1 introduces the evolution of object detection paradigms, and Section 2.2 discusses the specific challenges posed by UAV-based small object scenarios. Section 2.3 then compares the proposed STAIR-DETR with existing DETR-based detectors and explains the rationale behind our architectural choices. In addition, Table 1 provides a literature summary of several representative object detection algorithms.

### 2.1. Evolution of Object Detection Paradigms

Object detection has followed two principal paradigms: CNN-based and Transformer-based. Two-stage detectors (e.g., Faster R-CNN) achieve high accuracy via proposals, feature extraction, and classification/localization but entail prohibitive computational cost for real-time use; one-stage detectors (YOLO [18,19], SSD [20]) recast detection as direct regression to accelerate inference, yet their reliance on anchor boxes and NMS heightens hyperparameter sensitivity and undermines robustness to sparse or scale-variant targets. DETR introduced end-to-end set prediction, eliminating anchors and NMS but suffering from slow convergence, the expense of full self-attention, and weak small object representation in real-time UAV contexts; Deformable DETR mitigates these issues through sparse deformable attention. RT-DETR integrates efficient CNN backbones with Transformer encoders/decoders and incorporates layer-wise feature interaction and IoU-guided query selection, delivering a strong accuracy–latency trade-off. Accordingly, we adopt RT-DETR as the baseline and refine it to better preserve spatial detail and multi-scale representations for small objects in UAV imagery.

### 2.2. Challenges and Strategies for Small Object Detection in UAV Perspectives

UAV imagery predominantly contains small, low-resolution targets embedded in clutter, imposing challenges on feature representation and context modeling. Research addresses these issues through multi-scale representations, attention-based enhancement, and high-resolution feature utilization: FPN and its variants PANet and BiFPN aggregate cross-level semantics, and ASPP [21] enlarges the receptive field without reducing resolution, yet standard downsampling still discards fine details critical for precise localization. Attention modules such as SE-Net [22] and CBAM [23] improve discriminability by emphasizing informative channels or spatial regions, but primarily capture intra-map dependencies while overlooking distributional statistics that are crucial for highlighting weak object signals and suppressing background noise. Preserving shallow high-resolution features and attaching early detection heads [24,25] can compensate for detail loss in deeper layers; however, Transformer-based detectors like DETR decode on semantically rich yet spatially coarse features, rendering efficient integration of high-resolution detail without redundant computation a continuing challenge.

### 2.3. Improvements and Applications Based on the DETR Architecture

Research on the improvement and application of the RT-DETR model mainly focuses on query design, feature representation, and system-level coordination to enhance small target detection performance in drone imagery. Recent DETR-based variants generally improve small-target performance through multi-scale feature fusion, optimized query mechanisms, and fine-grained attention. For example, Deformable DETR [15] introduces sparse multi-scale self-attention to focus on critical regions, yet it still performs unsatisfactorily on extremely small objects. DN-DETR [26] and DINO [27] accelerate training convergence and boost recall of small targets by injecting noisy target queries and adopting cascade refinement. D-FINE [28] redefines bounding-box regression as a probabilistic distribution refinement process to improve precise localization of small objects. VRF-DETR [29] strengthens multi-scale representation by increasing receptive-field diversity and cross-scale feature interaction, improving sensitivity to scale variation in aerial scenes; however, its gains are limited when shallow high-resolution details are weakened or lost early. DFS-DETR [30] further enhances multi-scale supervision and query refinement to guide the detector toward small targets more effectively, but it still relies mainly on existing pyramid features and does not explicitly introduce higher-resolution prediction heads, leaving ultra-small objects vulnerable to detail attenuation during deep transformations. Although these improvements raise overall accuracy to some extent, they [31,32] share common drawbacks: extremely tiny objects remain difficult to detect reliably, and fixed downsampling and upsampling strategies in multi-scale fusion tend to discard fine details, making cross-scale consistency hard to guarantee.

However, existing models have issues that cannot be resolved by optimizing the decoder alone, such as insensitivity to small targets at shallow layers. Multi-scale fusion still limits performance improvement if it is not effectively matched with high-resolution prediction heads. Additionally, there is a problem of progressive loss of small target cues during deep feature transformation. Therefore, developing an end-to-end framework that jointly optimizes the backbone, neck, and decoder head is crucial for systematically mitigating the progressive loss of small-object cues during deep feature transformations, which is a key prerequisite for robust detection in UAV scenarios.

To address these existing issues, improvements will be made by constructing an end-to-end framework and jointly optimizing the backbone network, neck, and detection head to systematically alleviate the loss of small target cues. First, through DSEB (Dynamic Semantic Enhancement Block), multi-branch paths and dynamic convolution are integrated to enhance the semantic expression ability of the backbone network without sacrificing spatial precision, thereby enriching small target representations from the source. Second, SFA (Semantic Feature Aggregation) is used to replace the original AIFI in the neck, emphasizing fine-grained local target signals and suppressing background interference to improve sensitivity to small targets. Third, ASTO (Adaptive Scale Transformation Operator) unifies downsampling and upsampling into a context-aware scale transformation process, preserving fine details and cross-scale consistency, and reducing information loss caused by traditional scaling. Fourth, a high-resolution P2 detection head is added to directly utilize shallow features to enhance the sensitivity and localization accuracy of ultra-small targets.

**Table 1:** Comparative Summary of Representative Object Detectors.

Algorithm	References	Limitations	Environment Type	Convergence	Scalability
Faster R-CNN	[9], 2017	Incur high computational cost with slower inference than real-time detectors.	Suited to general detection that prioritizes accurate region proposals.	Improves via region proposal networks for stable, precise detection.	Robust in general scenarios but unsuitable for strict real-time or low-power deployments.
SSD	[20], 2016	Weak performance on small objects and inconsistent accuracy across scales.	Optimized for general-purpose detection with an emphasis on single-shot efficiency.	Stabilizes training through multi-scale feature fusion.	Applicable to general scenarios yet constrained in small object-dominant or highly complex environments.
YOLO	[18], 2016 [19], 2018 [33], 2025 [34], 2025	Fundamentally rely on CNN-based hierarchies and post-processing (NMS), weaker long-range dependency modeling compared to DETR.	Primarily designed for general real-time task	Benefiting from mature optimization strategies and dense supervision	It has strong adaptability to a variety of hardware platforms, but it has a bottleneck in dense occlusion scenes
DETR	[13], 2020 [15], 2021 [26], 2022 [27], 2022	Slow convergence; poor adaptation to small/occluded objects.	Aimed at end-to-end detection with minimal post-processing.	Deformable variants accelerate convergence and lower computational cost.	Suitable for general detection, yet constrained in small object and real-time applications.
RT-DETR	[16], 2023 [28], 2024 [29], 2025 [30], 2024 [31], 2022 [32], 2024 [35], 2024	Shows limited performance on small or aerial targets, and earlier versions may lag in real-time speed.	Developed to support real-time object detection across aerial, UAV, and remote-sensing applications.	Stabilizes accuracy through feature enhancement and variable receptive fields.	Delivers strong results on aerial and real-time tasks, yet capability declines in non-aerial or resource-constrained settings, it is difficult to ensure consistency across different scales.

Table 1 compares representative object detectors by contrasting their core designs, key reference literature, known limitations, deployment settings, convergence behavior, and scalability. The table shows that two-stage models face challenges in real-time performance, single-stage models are limited by sensitivity to small objects, and Transformer-based detectors are still balancing details and efficiency. These observations have motivated us to adopt high-resolution cues and cross-scale transformations to balance accuracy and speed.

## 3. Proposed Method

### 3.1. Overall Framework

**Figure 1:**
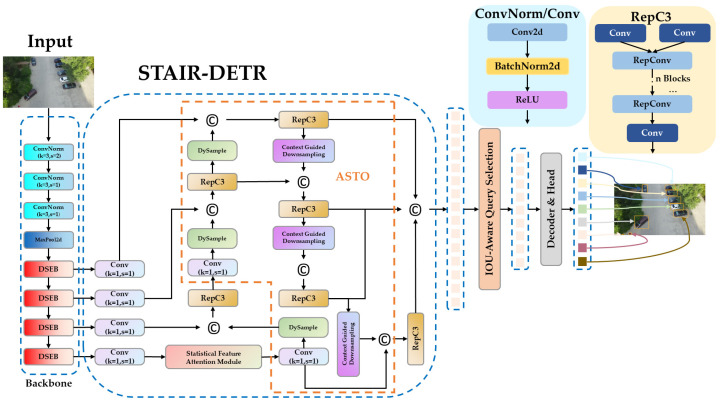
Overall architecture of STAIR-DETR.

To systematically address the challenge of progressive attenuation of small object information in deep neural networks from UAV viewpoints, an end-to-end synergistic detection model is proposed in the study, termed STAIR-DETR, which is built upon the RT-DETR framework. As illustrated in Figure 1, the framework integrates the backbone network, SFA mechanism, upsampling and downsampling modules, and the newly added P2 detection head to improve small object detection performance. The design philosophy of the proposed architecture is to reinforce the integrity and efficiency of information flow from feature extraction to final prediction.

The STAIR-DETR processing pipeline commences with the backbone network. The backbone stem follows RT-DETR, consisting of three Conv Norm layers (3 × 3, s = 1) and a MaxPool2d. The stride-1 Conv Norm stack keeps high-resolution shallow details for tiny UAV objects, and MaxPool2d then performs early down-sampling to enlarge the receptive field and reduce computation. This design retains fine-grained cues before deeper semantic extraction and multi-scale fusion. The DSEB module, a re-parameterizable module intended to boost feature variety at the source, is incorporated into the backbone to replace the residual units, thereby improving fine-grained feature representation while preserving computational efficiency. The SFA module is incorporated into the multi-scale fusion stage of the neck to accomplish effective global attention modeling with linear complexity, which improves the model’s capacity to differentiate small objects from visually comparable background areas.

An ASTO is shown to replace traditional downsampling and upsampling procedures for resolution alterations inside the feature pyramid. ASTO preserves spatial and semantic coherence across scales by adaptively restoring spatial details during upsampling and simultaneously preserving global context during downsampling. An additional P2 prediction head is integrated into the decoder to fully utilize the high-resolution features maintained throughout the network. This allows for direct prediction on shallow scale features and greatly increases the model’s sensitivity to small objects.

In the sections that follow, the previously indicated modules and their cooperative functions will be further explained.

### 3.2. Backbone Network Enhancement

In standard object detection frameworks, the backbone network typically comprises stacked residual modules such as BasicBlocks. A BasicBlock generally consists of two consecutive 3 × 3 convolutional layers, with residual connections facilitating information propagation across layers. Although this design ensures training stability and architectural simplicity, its fixed topology inherently limits the diversity of feature representations—particularly when facing complex backgrounds and multi-scale targets. In UAV-based tiny object detection scenarios, where targets are typically characterized by limited spatial extent, blurred boundaries, and weak textures, conventional residual blocks exhibit a constrained capacity to capture these critical local cues, thereby hindering detection accuracy.

**Figure 2:**
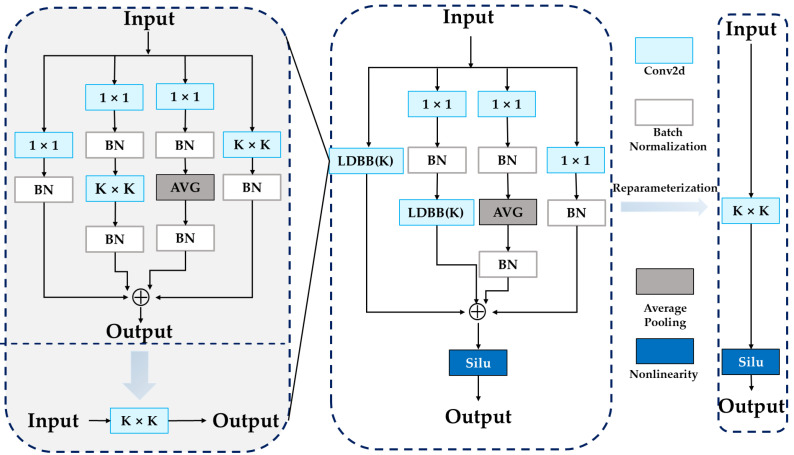
Structure of the Diverse Semantic Enhancement Block (DSEB).

Aiming to address these limitations, this study adopts the DSEB, a structurally re-parameterizable multi-branch module extending the fusion idea in YOLO-MIF [36]. This module enriches feature representation by combining multi-branch convolutions, with varied receptive fields, this ensures the retention of the fine-grained details that are crucial for the detection of small objects. Unlike YOLO-MIF’s mainly parallel convolutions, DSEB combines heterogeneous branches and merges them via structural re-parameterization, enabling diverse feature patterns during training while collapsing to a single 3 × 3 convolution at inference. Thus, feature expressiveness is increased without extra inference cost. DSEB is integrated into the STAIR-DETR backbone to strengthen representation capacity (Figure 2).

The training structure of DSEB includes the following five parallel branches:

(1) Main Branch: A standard 3 × 3 convolution capturing core local features;
(2) Multi-scale Branch: A 1 × 1 convolution enabling channel-wise interaction and fine-scale detail modeling;
(3) Sequential Convolution Branch: A composite of 1 × 1 followed by 3 × 3 convolutions, expanding the effective receptive field;
(4) Average Pooling Branch: Aggregates local statistical information, enhancing robustness to subtle geometric variations;
(5) Identity Mapping Branch: Preserves information flow through residual connections.

Outputs from all branches are summed element-wise and traverse a SiLU activation function. The SiLU function is defined as follows:

(1) $$SiLU(x)=x\cdot \sigma (x)=\frac{x}{1+e^{-x}}$$

In (1), x denotes the input value of the activation function (corresponding to each feature element after multi-branch summation). The term σ(x) represents the sigmoid function, formulated as $\sigma (x)=1/(1+e^{-x})$, which provides a smooth gating coefficient for the input. The constant e is the natural exponential base. The product x⋅σ(x) applies a soft and continuous modulation to the input, allowing SiLU to retain small negative responses while ensuring more stable gradients than ReLU—making it particularly suitable for multi-branch feature fusion scenarios.

After training, all uniform branches are re-parameterized into a single 3 × 3 convolution via the following steps:

(1) Batch normalization parameters are absorbed into preceding convolution weights and biases;
(2) 1 × 1 kernels are zero-padded to 3 × 3 dimensions;
(3) Average pooling is converted into a fixed-weight convolution kernel;
(4) Identity mapping is represented by a 3 × 3 identity kernel;
(5) All resulting kernels and biases are summed to produce a unified convolutional module.

To further enhance multi-scale representation, a lightweight variant LDSEB(k) uses parallel convolutions with different kernel sizes (e.g., k = 3, 5, 7). Each branch applies batch normalization and SiLU independently, then fuses via element-wise or weighted summation, with a residual path preserved. Larger kernels expand the receptive field to capture broader context while maintaining fine detail, improving the accuracy–robustness trade-off.

Embedded within the backbone, DSEB and LDSEB improve feature expressiveness without increasing model complexity or latency; in UAV settings, the enhanced backbone captures contours, textures, and local variations more reliably, improving detection under complex imaging conditions.

### 3.3. Enhancement of Feature Fusion Network

The Attention-in-Feature Interaction (AIFI) module, which is dominated by local convolution operations, is the main component that the Hybrid Encoder in RT-DETR uses to integrate multi-scale features that are taken from the backbone network. This architectural design constrains the capacity of the network to identify long-range dependencies across feature maps. In UAV imagery, distinguishing small targets from visually similar background distractors often requires a holistic understanding of the global scene. Consequently, it becomes essential to enhance the model with an efficient mechanism for global context modeling without incurring substantial computational overhead.

Accordingly, this research presents the Statistical Feature Attention (SFA) mechanism, an adaptation and extension of the Token-based Statistical Self-Attention (TSSA) [37] module. While TSSA leverages the statistical descriptors of feature tokens—such as mean and variance—to achieve lightweight global attention, SFA further refines this process through adaptive normalization and token weighting, enabling more effective global dependency modeling and improved small object discrimination in UAV detection tasks.

The core process of SFA includes three stages: statistical token extraction, variational attention estimation, and feature reweighting. First, the input feature map $F\in {\mathbb{R}}^{H\times W\times C}$ is unfolded into a 2D matrix $Z^{l}\in {\mathbb{R}}^{n\times d}$, where each n=H×W corresponds to a feature vector at a spatial position. Each attention head uses a projection matrix ${\left(U_{k}^{l}\right)}^{⊤}\in {\mathbb{R}}^{d\times p}$ to generate compressed features:

(2) $${\hat{Z}}_{k}={\left(U_{k}^{l}\right)}^{⊤}Z^{l}\in {\mathbb{R}}^{p\times n}$$

Next, SFA eschews pairwise similitude matrices. Instead, it constructs compact representations based on the statistical information (mean and standard deviation) of each token:

(3) $$\mu_{i}=\frac{1}{p}\sum_{j=1}^{p}{\hat{z}}_{i,j},\sigma_{i}^{2}=\frac{1}{p}\sum_{j=1}^{p}{\left({\hat{z}}_{i,j}-\mu_{i}\right)}^{2},s_{i}=[\mu_{i},\sigma_{i}]\in {\mathbb{R}}^{2}$$

Using the above statistics, SFA extracts channel-wise statistics (mean and standard deviation) for each token to form a statistical token $s_{i}=[\mu_{i},\sigma_{i}]$ as the input to the subsequent attention estimation. The attention coefficients are computed as:

(4) $$a_{i}=Sigmoid(W_{s_{i}}+b)$$

Here, $W\in {\mathbb{R}}^{1\times 2},b\in \mathbb{R}$ are learnable parameters. The Sigmoid function maps the attention values to the [0, 1] interval. The attention coefficients act on the input features in a diagonal fashion, meaning there is no need to construct a similarity matrix for n×n, thereby greatly reducing computational load. The reweighted output feature is:

(5) $$Z_{i}^{l+1}=a_{i}\cdot Z_{i}^{l}$$

This diagonal attention mechanism significantly reduces spatial and temporal complexity, respectively, O(p) and O(pn), compared to the standard self-attention’s $O(n^{2}d)$, making it better suited to managing problems involving sparse and high-resolution object detection in distant sensing photos.

The theoretical foundation of SFA is the MCR^2^ criterion, which promotes maximization of inter-cluster diversity and minimization of intra-cluster redundancy. After tokens are represented by statistical descriptors, attention weights are optimized under the following objective:

(6) $$\underset{U}{\min}\;\Delta R(Z^{l},\Pi )=\frac{1}{2}\log\;\det(I+\frac{\epsilon^{2}}{dn}Z^{l}{\left(Z^{l}\right)}^{⊤})-\frac{1}{2}\sum_{k=1}^{K}\frac{n_{k}}{n}\log\;\det(I+\frac{\epsilon^{2}}{dn_{k}}Z^{l}\mathrm{Diag}(\pi_{k}){\left(Z^{l}\right)}^{⊤})$$

where $\prod ={\left\{\pi_{k}\right\}}_{k=1}^{K}$ denotes the clustering of tokens (usually obtained via Gaussian Mixture Models or soft clustering), $n_{k}$ denotes the quantity of tokens in the *k*-th cluster, and ϵ is a constant adjustment parameter. The first term represents the covariance entropy of the entire token set, while the second term denotes the average coding rate within each cluster. Essentially, this objective maximizes inter-cluster divergence and minimizes intra-cluster redundancy, thereby improving the sensitivity of attention distribution to global structure.

**Figure 3:**
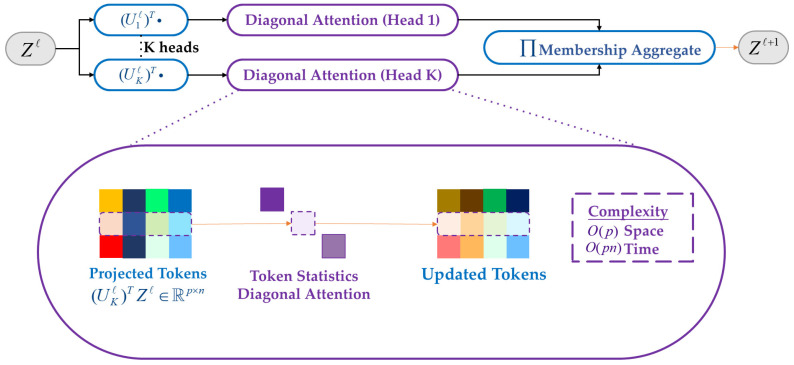
Illustration of the Statistical Feature Attention (SFA) mechanism.

Outputs from multiple attention heads are fused through the “Π Membership Aggregate” module, which aggregates the outputs of multiple attention heads based on the weights $\pi_{k}$ to obtain the final updated feature map $Z^{l+1}$. The overall framework of this process is shown in Figure 3.

For UAV-based tiny object detection, the Hybrid Encoder’s incorporation of SFA offers several benefits. The diagonal attention design, guided by token-level statistical features and multi-head aggregation, reduces computational cost while enhancing discriminability of small objects. A global receptive field with linear time complexity is introduced, the module enables efficient modeling of long-range dependencies that are crucial for distinguishing small objects from background clutter. At the same time, its foundation in information theory ensures that the learned attention weights remain theoretically grounded and interpretable, which enhances the reliability of the representation. Furthermore, as a lightweight and computationally efficient operator, SFA can be seamlessly included in real-time detection frameworks, improving accuracy while maintaining inference efficiency.

### 3.4. Adaptive Scale Transformation Operators

Conventional Feature Pyramid Networks (FPNs) use fixed operators for scale transformations: strided convolutions for downsampling and nearest-neighbor or bilinear interpolation for upsampling. These static methods fail to adapt to feature content, causing loss of shallow-layer small object details during downsampling and blurred upsampled features in upsampling pathways. These information bottlenecks during scale transformation significantly restrict the detection performance of modern detectors.

**Figure 4:**
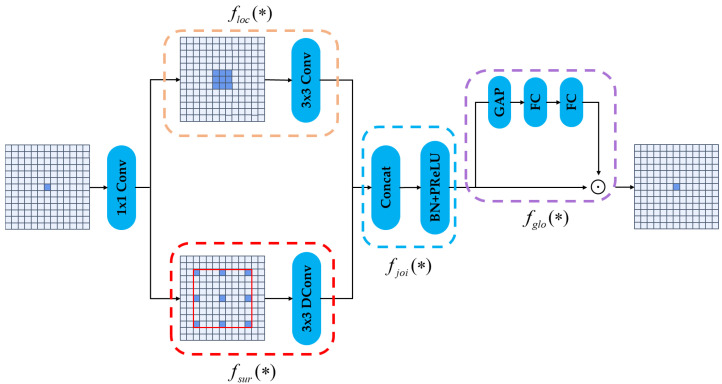
Architecture of the feature fusion block.

**Figure 5:**
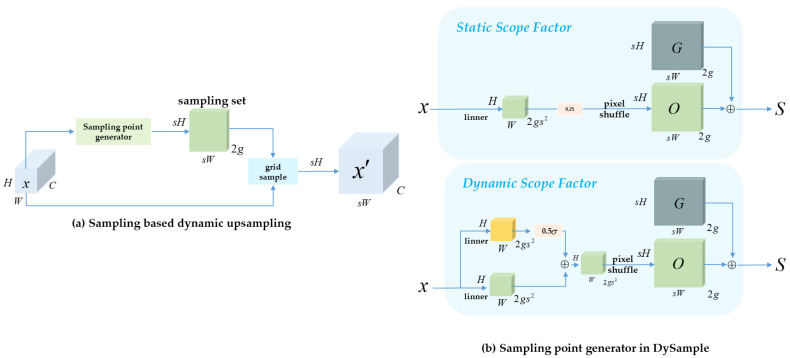
The architecture diagram of DySample.

**Figure 6:**
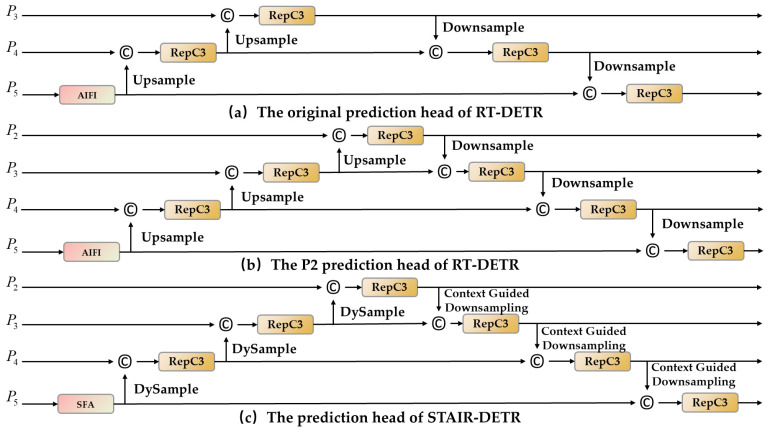
A comparison of prediction heads in RT-DETR and STAIR-DETR.

This study introduces the ASTO, combining Context-Guided Downsampling (CGD) [38] for bottom-up paths and Dynamic Sampling (DySample) [39] for top-down paths. This forms a context- and content-adaptive strategy that dynamically adjusts based on input features, as illustrated in Figure 4, Figure 5 and Figure 6.

In the bottom-up path, ASTO replaces standard strided convolutions with CGD, which incorporates a parallel gating mechanism alongside the main convolutional branch, as illustrated in Figure 4. CGD fuses local perception, surrounding context, and global channel attention to guide the filtering and compression of features during downsampling, thereby enhancing the discriminative power of spatial structures.

The diagram shows the integration of multiple convolutional and fully connected layers, including 1 × 1 convolutions, 3 × 3 convolutions, depth wise convolutions (3 × 3 DConv), and global average pooling (GAP) operations.

Specifically:

$f_{loc}(*)$ represents the local information extraction branch, It captures local neighborhood information focused at each spatial point using a typical 3 × 3 convolution. This process is mathematically expressed as:

(7) $$f_{loc}(x)=Conv_{3\times 3}(x)$$

$f_{sur}(*)$ denotes the context-aware branch, which uses a 3 × 3 dilated convolution to provide a wider spatial context and expand the receptive field:

(8) $$f_{sur}(x)={\mathrm{DilatedConv}}_{3\times 3}{,}_{r}(x)$$

$f_{joi}(*)$ corresponds to the feature fusion and nonlinear mapping branch, where BN + PReLU is used for nonlinear transformation after local and contextual features are concatenated along the channel dimension:

(9) $$f_{joi}(x)=\mathrm{BN}+\mathrm{PReLU}(\mathrm{Concat}(f_{loc},f_{sur}))$$

$f_{glo}(*)$ indicates the global channel guidance branch, inspired by the SE module. After extracting global semantics using global average pooling (GAP), two fully connected (FC) layers are used to create a channel attention vector:

(10) $$f_{glo}(x)=\sigma ({\mathrm{FC}}_{2}{(\mathrm{ReLU}(\mathrm{FC}}_{1}(\mathrm{GAP}(f_{joi})))))$$

Finally, the outputs from $f_{joi}$ and $f_{glo}$ are combined through channel-wise multiplication to generate the final output:

(11) $$f_{out}=f_{joi}⊙f_{glo}$$

where ⊙ denotes element-wise multiplication across the channel dimension. This mechanism selectively amplifies object-relevant features before compression, significantly enhancing small object representation in deeper layers. It ensures that the compression process is both spatially and channel-wise adaptive, effectively preserving discriminative cues for small object detection.

In the top-down path, ASTO replaces fixed interpolation with DySample, which generates content-adaptive upsampling kernels at each spatial location. The two parts of DySample are content-aware up sampling and dynamic kernel generation, as shown in Figure 5a: dynamic kernel generation and content-aware up sampling. First, the input feature map passes through a linear layer to generate sampling offsets $O\in {\mathbb{R}}^{sH\times sW\times 2g}$, where the number of sampling points per output location is indicated by g, and s denotes the upsampling ratio. Given a standard sampling grid G, the final set of offset sampling points is defined as:

(12) S=G+λ(x)⋅O

Here, λ(x) denotes the dynamic range factor, predicted by an auxiliary branch with a sigmoid activation:

(13) λ(x)=0.5⋅σ(W3x)

The final feature resampling is performed using the grid sample operation based on bilinear interpolation:

(14) X′=grid_sample(X,S)

The output has a resolution of $X^{\prime }\in {\mathbb{R}}^{C\times sH\times sW}$, and supports arbitrary up sampling scales such as 2× or 4×. DySample adaptively adjusts the spatial distribution and density of sampling points based on image content, enabling more precise restoration of details and textures—particularly effective for reconstructing small object edges and structures.

Figure 5b illustrates the sampling point generation mechanism of DySample, supporting two strategies for the sampling range factor: static and dynamic. In the static strategy, the offset field O is generated via a linear transformation of the input features, multiplied by a constant factor (e.g., 0.25), and added to the base grid G to produce the sampling set S. This method applies a uniform sampling range across all spatial locations. The dynamic scope factor adapts to varying input resolutions and network conditions, improving the spatial distribution of sampling points.

In contrast, the dynamic strategy first predicts a location-specific range factor λ(x)=0.5⋅σ(linear(x)), through an auxiliary branch, which is then multiplied by the offset field. This allows different regions to determine their own “sampling aggressiveness” based on content, enabling fine-grained control over reconstruction. Compared with the static approach, this dynamic strategy better captures texture, edges, and other local salient details. Although DySample introduces additional computation compared to static interpolation, its lightweight kernel predictor ensures that the overhead remains acceptable for real-time detection tasks.

ASTO replaces the original FPN with stacked modules: CGD for bottom-up compression and DySample for top-down restoration. This unified framework enables cross-scale adaptive reconstruction, enhancing cue preservation and detection performance in STAIR-DETR, particularly for small objects in complex UAV environments.

### 3.5. STAIR-DETR Detection Head

This study addresses the omission of P2 high-resolution features in the original RT-DETR head, which concentrates object queries and predictions on P3 to P5 to balance accuracy and latency but compresses and blurs P2 information when routed through lateral connections, thereby weakening small object representation. We incorporate a dedicated P2 prediction branch into the RT-DETR decoder that operates in parallel from P3 to P5 while sharing decoder layers for parameter efficiency; the branch receives its own object queries and a specialized prediction subhead, enabling direct exploitation of high-resolution features for precise localization and classification in UAV scenarios dominated by small objects.

As shown in Figure 6, our design adds the P2 prediction head while simultaneously replacing the original Upsample, Downsample and AIFI modules with DySample, Context-Guided Downsampling, and SFA, resulting in the final STAIR-DETR prediction head shown in Figure 6c. These improvements collectively enhance the model ability to detect small UAV targets with higher accuracy and robustness.

Concretely, the shallow feature map can be represented as:

(15) $$F_{P2}\in {\mathbb{R}}^{H\times W\times d}$$

It is first projected via a linear transformation to produce key-value pairs:

(16) $$K_{P2},V_{P2}=Linear(F_{P2})$$

which are then combined with the P2-specific object queries $Q_{P2}$ and input into the decoder interaction module:

(17) $$O_{P2}=Decoder(Q_{P2},K_{P2},V_{P2})$$

The decoder output $O_{P2}$ is subsequently processed by the prediction head to yield category probabilities and bounding box coordinates:

(18) $$P_{cls}^{\left(P2\right)}=Softmax(W_{cls}\cdot O_{P2}+b_{cls})$$

(19) $$B^{\left(P2\right)}=\sigma (W_{reg}\cdot O_{P2}+b_{reg})$$

The output from this P2 branch is optimized jointly with the outputs from P3–P5 via Hungarian matching and end-to-end loss computation.

This design integrates high-resolution cues with minimal computational overhead, enhancing model sensitivity to small-scale targets. By adding the P2 branch, the model effectively exploits low-level, detail-rich features, improving small object recall and precision. Relative to original RT-DETR, the proposed model not only includes P2 features but also employs DySample and Context-Guided Downsampling to strengthen contextual modeling and cross-scale flow. As seen in Figure 6c, the final STAIR-DETR detection head adopts a multi-scale, multi-path fusion strategy, integrating semantic and structural cues from multiple levels and using the RepC3 module for refined feature encoding. This yields substantial detection improvements, especially for small objects. Detailed experimental validations in Section 4.3 demonstrate the approach’s effectiveness and generalizability in real-world scenarios.

## 4. Experimental Results and Analysis

### 4.1. Dataset and Experimental Setup

#### 4.1.1. Dataset

This study adopts the VisDrone2019 dataset, a widely recognized UAV aerial imagery benchmark published by the Data Mining and Machine Learning Lab at Tianjin University, as the basis for model training and evaluation. There are 1610 test images, 548 validation images, and 6471 training images in the dataset, collected from diverse urban and rural locations across China under varying illumination and weather conditions, including daytime, nighttime, sunny, and overcast scenarios. Such variability poses substantial challenges for object detection models in terms of generalization and robustness. VisDrone2019 defines ten object categories, such as tricycle, awning-tricycle, motorbike, bicycle, truck, van, bus, pedestrian, and people, and car, and is characterized by small object sizes, dense spatial distributions, and severe occlusion due to the top-down UAV perspective. These characteristics impose stringent requirements on multi-class recognition, scale adaptability, and detection stability.

In addition, to examine whether our scale-related designs remain consistently effective across different aerial benchmarks, we conduct cross-dataset supplementary experiments on the public DOTA-v1.0 dataset [40]. DOTA-v1.0, released by a research team from Wuhan University, is a widely used aerial remote-sensing detection benchmark. It contains 188,282 annotated instances, each labeled by an arbitrary quadrilateral (oriented bounding box). Following the experimental setting in this paper, DOTA-v1.0 is organized into 15,749 training images and 5297 validation images. The dataset features complex scenes and wide scale variation, and covers fifteen categories: small-vehicle, large-vehicle, plane, storage-tank, ship, harbor, ground-track-field, soccer-ball-field, tennis-court, swimming-pool, baseball-diamond, roundabout, basketball-court, bridge, and helicopter. Since the test-set annotations are not publicly available, we report results on the validation split. This supplementary evaluation helps verify that the performance gains of our method across object scale are consistent and generalizable beyond VisDrone.

#### 4.1.2. Data Augmentation Strategy

To enhance generalization under these complex conditions and mitigate overfitting, during training, several online augmentation techniques are used. Geometric adjustments like cropping, random flipping, and scaling are first employed to diversify spatial distributions and object scales. Mosaic augmentation is further incorporated to merge four images into one, thereby enriching background variability and amplifying small object density. In addition, perturbations in the color space of HSV, through adjustments of hue, saturation, and brightness simulate illumination variations and improve robustness against color discrepancies. Taken together, these augmentation techniques greatly increase the variety of the training data and provide a solid foundation for trustworthy model learning.

#### 4.1.3. Experimental Environment and Configuration

The proposed STAIR-DETR model is developed on the publicly available RT-DETR-r18 framework with targeted architectural enhancements. For comprehensive comparison the enhanced STAIR-DETR and the baseline RT-DETR are trained and assessed under the same conditions. On a server with an NVIDIA RTX 4080 Super GPU, the experiments are conducted using Python 3.8 and the PyTorch 2.4.1 module. The model is optimized using the AdamW algorithm, which has an initial learning rate of 0.0001, a final rate of 1, and a weight decay of 0.0001. To facilitate convergence, a cosine annealing scheduler is used, and training is carried out for 200 epochs with a batch size of four.

### 4.2. Evaluation Metrics

Standard detection criteria such as Precision, Recall, FPS and mean Average Precision (mAP) are used to evaluate the suggested model, and GFLOPs is used to gauge computational efficiency.

Recall calculates the percentage of actual positives that were properly recognized, whereas precision counts the percentage of positive samples that were accurately predicted:

(20) $$Precision=\frac{TP}{TP+FP}$$

(21) $$Recall=\frac{TP}{TP+FN}$$

Here, TP (True Positives) indicates the correctly identified objects, FP (False Positives) refers to misclassified background regions, and FN (False Negatives) corresponds to ground-truth instances that were not detected.

For each object class, Average Precision (AP) is calculated by taking the area under the Precision-Recall(P-R) curve that corresponds to it.

(22) $$AP=\int_{0}^{1}P(R)dR$$

The overall performance is summarized by mean Average Precision (mAP):

(23) $$mAP=\frac{1}{C}\sum_{c=1}^{C}AP_{c}$$

where the number of categories is represented by *C*. This study reports mAP50 (IoU = 0.5) and mAP50:95 (averaged over IoU thresholds from 0.5 to 0.95 using step 0.05).

GFLOPs, which is the number of floating-point operations required for a single forward pass normalized to billions, is a measure of computational complexity:

(24) $$GFLOPS=\frac{FLOPs}{10^{9}}$$

This metric provides a hardware-independent indicator of computational cost.

In addition, Frames Per Second (FPS) is used to characterize inference throughput, that is, the number of images the model can process per unit time during evaluation. FPS is computed as:

(25) $$FPS=\frac{N}{T}$$

where N denotes the total number of images processed and T is the corresponding inference time in seconds. A higher FPS indicates a faster model and better real-time processing capability.

### 4.3. Ablation Experiments

To determine the contribution of each component within the STAIR-DETR framework, this study conducted ablation experiments on the VisDrone dataset using RT-DETR-r18 as the baseline. First, we integrated DSEB into the backbone to strengthen feature abstraction and representation. Next, we integrated SFA into the neck within the AIFI stage to model global context more effectively. Then, we adopted ASTO in place of conventional convolution downsampling and upsampling. ASTO combines context-aware downsampling and content-adaptive upsampling to keep multi-scale details and to enhance contextual learning. We also added a P2 prediction branch in the detection head to use high-resolution shallow features and to improve small object detection under clutter and scale variation.

The results are shown in Table 2. Each addition brings about a continuous increase. The baseline reaches an mAP50 of 36.2 percent with a model size of 20.2 MB. After integrating DSEB and SFA, accuracy rises, and DSEB in the backbone lifts mAP50 to 38.0 percent. Adding the P2 branch increases mAP50 to 40.2 percent, which shows the value of high-resolution shallow features. With DSEB, SFA, and the P2 head together, mAP50 is 40.7 percent, mAP50:95 is 24.3 percent, and the size drops to 18.4 MB. Integrating ASTO brings further gains. The context-aware downsampling improves recognition with only a small size increase. The adaptive upsampling restores fine details and gives the best mAP50 of 41.7 percent while keeping mAP50:95 at 23.4 percent. The cost remains controlled at 86.6 GFLOPs, and the total size grows by about 1.0 MB compared with the baseline, which is about a 5.4 percent increase in parameters. At the same time, the ablation study shows that as modules are added progressively, the FPS value gradually decreases and eventually drops to 40.2, yet the model ultimately achieves a substantial improvement in detection accuracy. Overall, the combination of DSEB, SFA, ASTO, and the P2 head gives clear improvements on VisDrone and supports the practical value of each component.

### 4.4. Performance Comparison

To evaluate the efficacy and competitiveness of STAIR-DETR, we conduct a comprehensive comparison with representative two-stage, one-stage, and end-to-end detectors on the VisDrone benchmark and further assess generalization on the DOTA-v1.0 benchmark (DOTA-v1.0). The test results on the VisDrone test set are shown in Table 3, STAIR-DETR achieves a mAP50 of 41.7% and an mAP50:95 of 23.4%, outperforming the baseline RT-DETR-r18 by 4.9 and 2.7 percentage points, respectively. It also surpasses the strongest competing end-to-end methods DFS-DETR and VRF-DETR, improving mAP50 by 1.0 and 1.8 percentage points, while attaining a slightly higher mAP50:95 than VRF-DETR. These results confirm that the proposed architectural refinements effectively improve small object detection accuracy and localization precision in complex UAV imagery.

From a computational perspective, STAIR-DETR achieves a good balance between accuracy and efficiency. The model operates at 86.6 GFLOPs, which is lower than computationally more demanding frameworks such as Deformable DETR and Faster R-CNN, but slightly higher than some lightweight detectors. The parameter count is 21.2 M and increases by only 4.9% compared with the baseline model, maintaining moderate complexity and supporting effective training and practical deployment. These increases in complexity arise from the additional feature refinement and multi-scale modeling modules that are specifically introduced to improve the accuracy of small object detection. These components deepen the feature hierarchy and increase the amount of transformer computation, which leads to a certain reduction in inference speed but also brings a substantial improvement in detection quality. Overall, on the VisDrone dataset, the proposed method exhibits clear advantages in detection accuracy, localization precision, and efficiency, confirming the robustness and applicability of the approach for UAV small object detection in complex environments.

Notably, STAIR-DETR demonstrates significant gains over the baseline across all object sizes on the DOTA and VisDrone benchmarks—small, medium, and large—with medium-scale objects showing the greatest improvement. The test results are shown in Table 4. Specifically, the mean Average Precision for medium objects mAP_m reaches 56.3%, representing a 12.3% increase over the baseline. For small objects mAP_s, the score improves to 28.2%, up by 7.1%, while for large objects mAP_l, it achieves 60.5%, a gain of 8.9%. On VisDrone, compared to the baseline model, the average precision for large, medium, and small-sized objects has also increased by 6.2%, 4.2%, and 17.6%, respectively.

These results underscore STAIR-DETR’s ability to accurately detect objects at diverse scales, especially small and medium instances within challenging aerial scenes.

Compared with alternative SOTA models, STAIR-DETR continues to exhibit a distinct performance lead. It is 9.0 percentage points higher than the next-best competitor VRF-DETR (47.3%) on mAP_m, which further demonstrates the significant value of its architectural innovations in detecting objects across scales.

Taken together, the improvements observed in mAP50, mAP50:95, and scale-specific metrics mAP_s, mAP_m, mAP_l highlight the impact of the architectural enhancements introduced in this work. Enhancements to the backbone, neck, and detection head jointly drive substantial accuracy gains across object sizes, affirming STAIR-DETR’s robustness and adaptability in practical detection tasks.

### 4.5. Visualization Analysis

**Figure 7:**
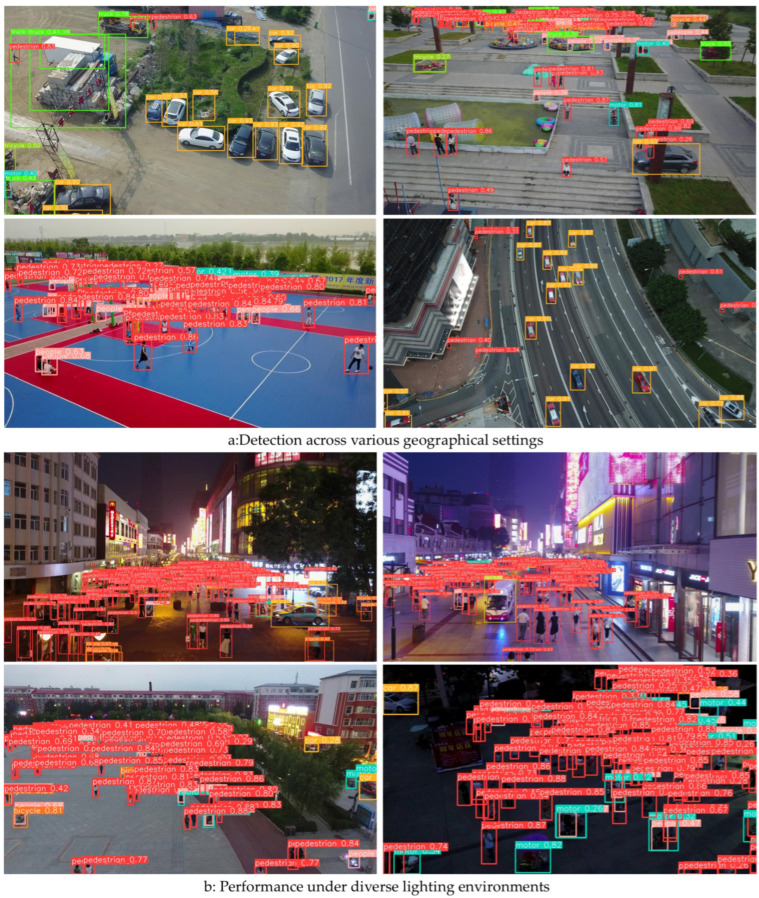

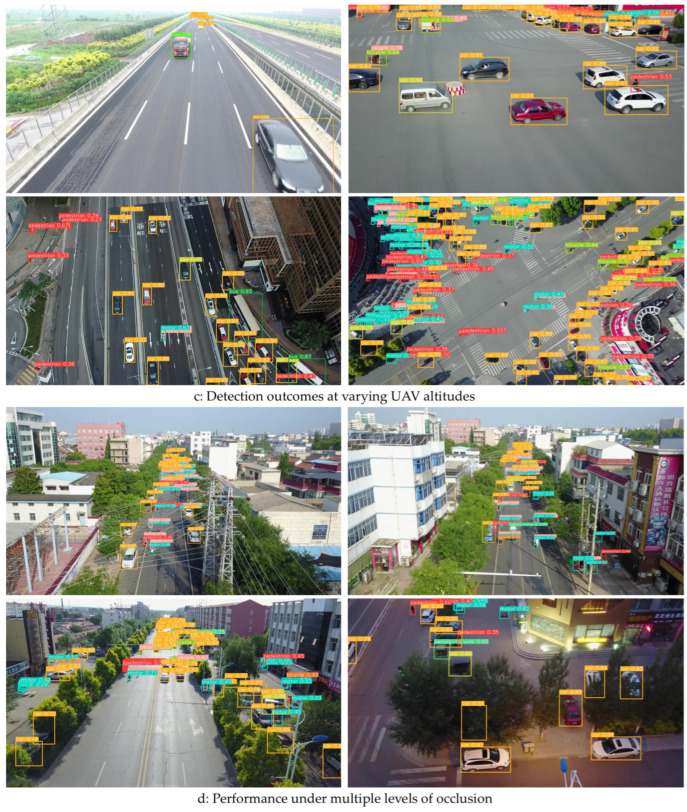
The visualization detection effects of models in various complex environments.

Small object detection often suffers from environmental interference, such as cluttered backgrounds, varying lighting conditions, changes in viewing angles, and partial occlusion. To evaluate the real-world robustness of STAIR-DETR, this study visualizes detection results across representative challenging scenarios, as depicted in Figure 7.

The visual results underscore the model’s generalization capability across diverse environmental conditions. For instance, despite changes in background complexity or scene type, STAIR-DETR maintains reliable detection outputs, indicating its resilience to spatial and contextual variation. Under poor lighting conditions, including dim or low-visibility environments, the model is still able to detect key targets with high localization accuracy. Moreover, as the UAV altitude increases, reducing object scale and increasing occlusion—STAIR-DETR remains responsive to the essential features in the image, reflecting its robustness to scale changes and perspective variation. Although some missed detections are observed in highly occluded areas, the model successfully identifies most instances, suggesting a certain level of occlusion tolerance, though room for future improvement remains.

**Figure 8:**
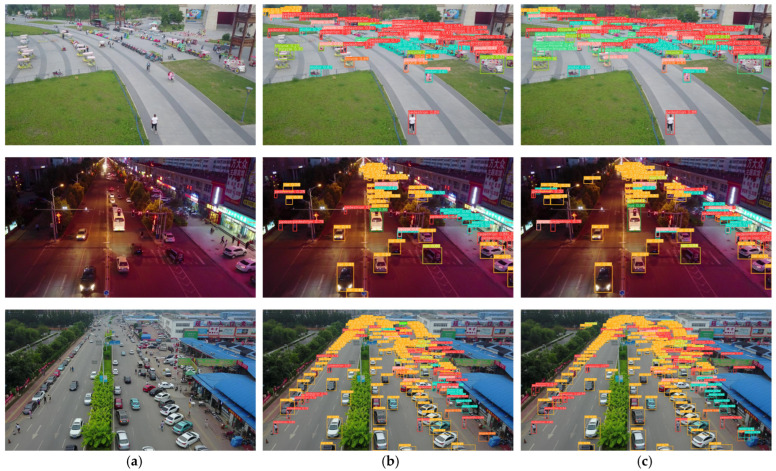
Visual comparison of detection results between RT-DETR-r18 and STAIR-DETR. Subfigure (**a**) shows the input image, (**b**) shows predictions by RT-DETR-r18, and (**c**) shows outputs from STAIR-DETR.

Figure 8 illustrates a comparative visualization between STAIR-DETR and the RT-DETR-r18 baseline under a range of real-world scenarios. The comparison clearly demonstrates that STAIR-DETR delivers superior detection accuracy and stability compared to the baseline. In challenging settings such as nighttime scenes or dense urban areas, the baseline model tends to produce false positives or overlook small-scale targets. In contrast, STAIR-DETR consistently identifies relevant objects—including small vehicles and pedestrians—while reducing misclassification, and assigns higher confidence to true positives.

**Figure 9:**
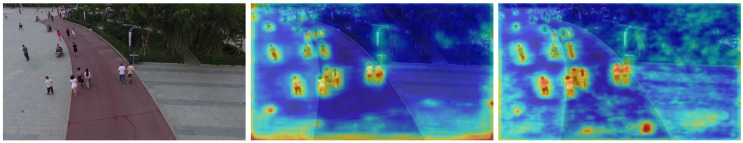

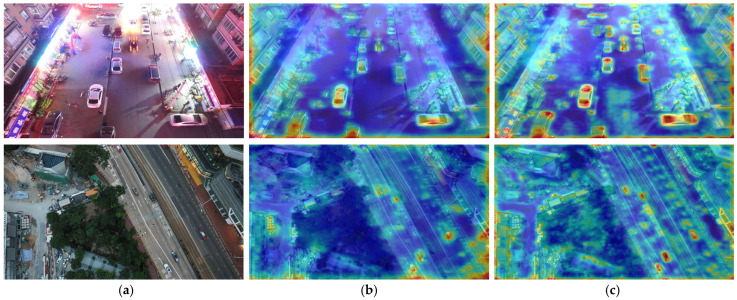
Analysis of feature maps on the VisDrone-test dataset. Figure (**a**) is the original image, figure (**b**) is the heatmap image on RT-DETR, and figure (**c**) is the heatmap of our STAIR-DETR.

To further interpret the model’s focus on relevant regions, this study employs Grad-CAM++ for visualizing feature activations (see Figure 9). This technique enables more precise interpretation, especially in scenes with overlapping targets. The heatmaps reveal that STAIR-DETR allocates its attention more precisely to target areas, as indicated by the intensity of activation in key regions. Compared to RT-DETR, which shows more dispersed and less focused attention, STAIR-DETR demonstrates superior capacity to capture meaningful features and suppress background noise. This reinforces its effectiveness in scenarios with small or densely packed objects.

In addition, we analyze false positives and missed detections using heatmaps and qualitative visualizations. The results show that most errors occur in extremely crowded regions with severe occlusions, or when the object size is smaller than 4 × 4 pixels. In these situations, even the enhanced backbone and P2 head struggle to extract sufficiently discriminative cues, leading to missed detections or inaccurate localization. A small portion of false positives also arises from background structures, such as building edges and road markings, which resemble distant vehicles. These observations are consistent with the intrinsic limitations of purely appearance-based detectors and motivate future extensions that exploit temporal cues or multimodal information.

## 5. Discussion

This work shows that for UAV-based tiny object detection, STAIR-DETR consistently improves detection accuracy and computational efficiency. The proposed modules, including the Statistical Feature Attention (SFA), Diverse Semantic Enhancement Block (DSEB), Adaptive Scale Transformation Operator (ASTO), and P2 detection head, are validated through systematic ablation experiments. DSEB enhances semantic representation through parallel multi-scale branches with dynamic convolution while preserving spatial fidelity. SFA constructs adaptive attention distributions based on statistical feature properties, allowing the model to reduce background noise and highlight key areas in noisy UAV imagery. ASTO unifies resolution transformation by combining context-guided downsampling with dynamic upsampling, reducing semantic loss and restoring structural detail, while the P2 head leverages high-resolution shallow features to enhance the detection and localization of extremely small targets. Comparative assessments against established detectors including Faster R-CNN, YOLO series, RT-DETR variants, and VRF-DETR confirm the superiority of STAIR-DETR in mean average precision while maintaining real-time inference capability. The model also exhibits strong robustness under diverse illumination conditions, dense object distributions, and severe occlusions.

Although STAIR-DETR achieves high accuracy and robustness, the architectural complexity introduced by SFA and ASTO may constrain deployment on computationally constrained UAV platforms. Future work should explore model compression strategies such as quantization, pruning and knowledge distillation to effectively reduce inference overhead. Incorporating temporal cues or multimodal data, including infrared and LiDAR, may further enhance adaptability for dynamic and safety-critical UAV missions. These directions will enable the continued evolution of STAIR-DETR toward efficient, reliable, and deployable real-world applications.

## 6. Conclusions

This study presents STAIR-DETR, a UAV-oriented detection framework that systematically enhances feature extraction, feature fusion, and resolution transformation to address the difficulties in small object detection in aerial imagery. By introducing architectural refinements across multiple stages of the network, STAIR-DETR achieves superior detection accuracy and localization precision while maintaining real-time inference efficiency. Experimental results on the VisDrone2019 benchmark confirm its effectiveness, with the model attaining 41.7% mAP@50 and 23.4% mAP@50:95, outperforming the baseline RT-DETR and several state-of-the-art detectors. These results highlight the robustness of the framework under complex UAV imaging conditions, including cluttered backgrounds, occlusion, and scale variation. Looking ahead, future research will focus on enhancing deployment efficiency through lightweight model designs and extending the framework to incorporate multimodal and temporal information, thereby broadening its applicability to diverse and dynamic UAV-based perception tasks.

## Figures and Tables

**Table 2:** Ablation experiments comparison results.

DSEB	SFA	P2	CGD	DySample	mAP50 (%) ↑	mAP50:95 (%) ↑	FLOPs (G) ↓	Param (M) ↓	FPS ↑
					36.2	20.7	57.0	20.2	103.0
√					38.0	22.0	57.0	19.8	100.7
	√				37.4	21.8	57.1	19.7	107.2
√		√			40.2	23.9	78.2	18.6	70.1
	√	√			39.8	23.7	78.3	18.4	72.9
√	√	√			40.7	24.3	78.3	18.4	72.6
√	√	√	√		41.2	23.2	84.8	21.0	66.1
√	√	√	√	√	41.7	23.4	86.6	21.2	40.2

Note: ↑ indicates that a higher value is better; ↓indicates that a lower value is better; √ indicates that the corresponding module is enabled.

**Table 3:** The detection results of the proposed model compared with the SOTA models on the VisDrone test dataset.

Models	mAP50 (%) ↑	mAP50:95 (%) ↑	FLOPs (G) ↓	Param (M) ↓	FPS ↑
*Two-stage methods*					
Faster R-CNN [9]	32.3	12.0	127.0	41.7	36.2
Cascade-R-CNN [41]	32.8	19.5	236	69.4	31.6
*One-stage methods*					
YOLOv8m [42]	33.2	19.0	79.8	25.9	256.0
YOLOv11m [43]	35.7	20.6	59.0	15.3	752.8
YOLOv12m [44]	31.7	18.3	67.5	20.2	122.4
YOLOv13n [45]	26.3	14.5	6.1	2.5	264.3
S-YOLO [33]	36.4	21.3	25.3	2.7	285.0
DR-YOLO [34]	38.5	20.9	8.1	3.1	57.8
*End-to-end methods*					
Deformable DETR [15]	30.2	16.4	172.5	40.2	19
RT-DETR-r18 [16]	36.8	20.7	57.0	20.2	103.0
RT-DETRv2-r18 [35]	39.1	22.1	60.0	20.0	-
D-Fine-M [28]	39.6	22.1	56.4	19.2	480.9
VRF-DETR [29]	39.9	23.3	44.6	13.8	158.7
DFS-DETR [30]	40.7	-	-	19.8	-
ours	41.7	23.4	86.6	21.2	40.2

Note: ↑ indicates that a higher value is better; ↓indicates that a lower value is better.

**Table 4:** The detection comparison results of proposed model on the DOTAv1.0-val and VisDrone-test dataset.

Dataset	Models	mAP_s (%) ↑	mAP_m (%) ↑	mAP_l (%) ↑	mAP50 (%) ↑	mAP5:95 (%) ↑
DOTA	RT-DETR-r18 [16]	21.1	44	51.6	65.1	41.7
	VRF-DETR [36]	23.2	47.3	53.8	68.6	43.8
	ours	28.2	56.3	60.5	69.7	43.4
VisDrone	RT-DETR-r18 [16]	9.1	26.3	33.7	36.2	20.7
	ours	10.7	27.4	35.8	41.7	23.4

Note: ↑ indicates that a higher value is better.

## Data Availability

The VisDrone datasets: https://github.com/VisDrone/VisDrone-Dataset, accessed on 8 September 2025; The DOTAv1.0 datasets: https://www.modelscope.cn/datasets/yolo_master/DOTAv1/files, accessed on 8 December 2025. The original contributions presented in this study are included in the article. Further inquiries can be directed to the corresponding author.
